# Supplementary material for: Is there a competitive advantage to using multivariate statistical or machine learning methods over the Bross formula in the hdPS framework for bias and variance estimation?
Source: PLoS One. 2025 May 28;20(5):e0324639. doi: 10.1371/journal.pone.0324639 (PMC12118903; doi:10.1371/journal.pone.0324639)
Supplement: S1 File — (PDF) [file pone.0324639.s001.pdf]

Supplementary Content

Is There a Competitive Advantage to Using Multivariate Statistical or Machine Learning Methods Over the Bross Formula in the hdPS Framework for Bias and Variance Estimation?

A Variables Used for Plasmode Simulation Data Generation

1. Original demographic variables (8)

- age,
- sex,
- education,
- race,
- marital status,
- income,
- country where born,
- survey cycle

2. Original behaviour variables (5)

- smoking,
- diet,
- high cholesterol,
- physical activity,
- sleep

3. Original health history / access variables (2)

- diabetes family history,
- medical access

4. Transformed lab variables (6) (complex forms) based on original lab variables: uric acid, protein, bilirubin, phosphorus, sodium, potassium, globulin, calcium, systolic blood pressure, diastolic blood pressure.

- Tranfored.var.1 = log(globulin)
- Tranfored.var.2 = protein*calcium
- Tranfored.var.3 = diastolicBP/systolicBP)^2
- Tranfored.var.4 = sqrt(uric acid+bilirubin)/2
- Tranfored.var.5 = phosphorus^2/(sodium*potassium)
- Tranfored.var.6 = log(systolicBP+10)

5. Count based prescription codes (1) (proxies of comorbidity)

Simple count (1 variable) = sum of selected ICD-10 CM codes (converted to recurrence covariates) who had less than 0.8 or greater than 1.2 compared to the outcome = $\sum_{s=1}^{94} R_s$

B True Outcome Model for Plasmode Simulation Data Generation

Diabetes (outcome) = Obese (exposure) + demographic/behaviour/health history variables + transformed lab variables + simple count with selected ICD-10 codes

C Standard Error comparison

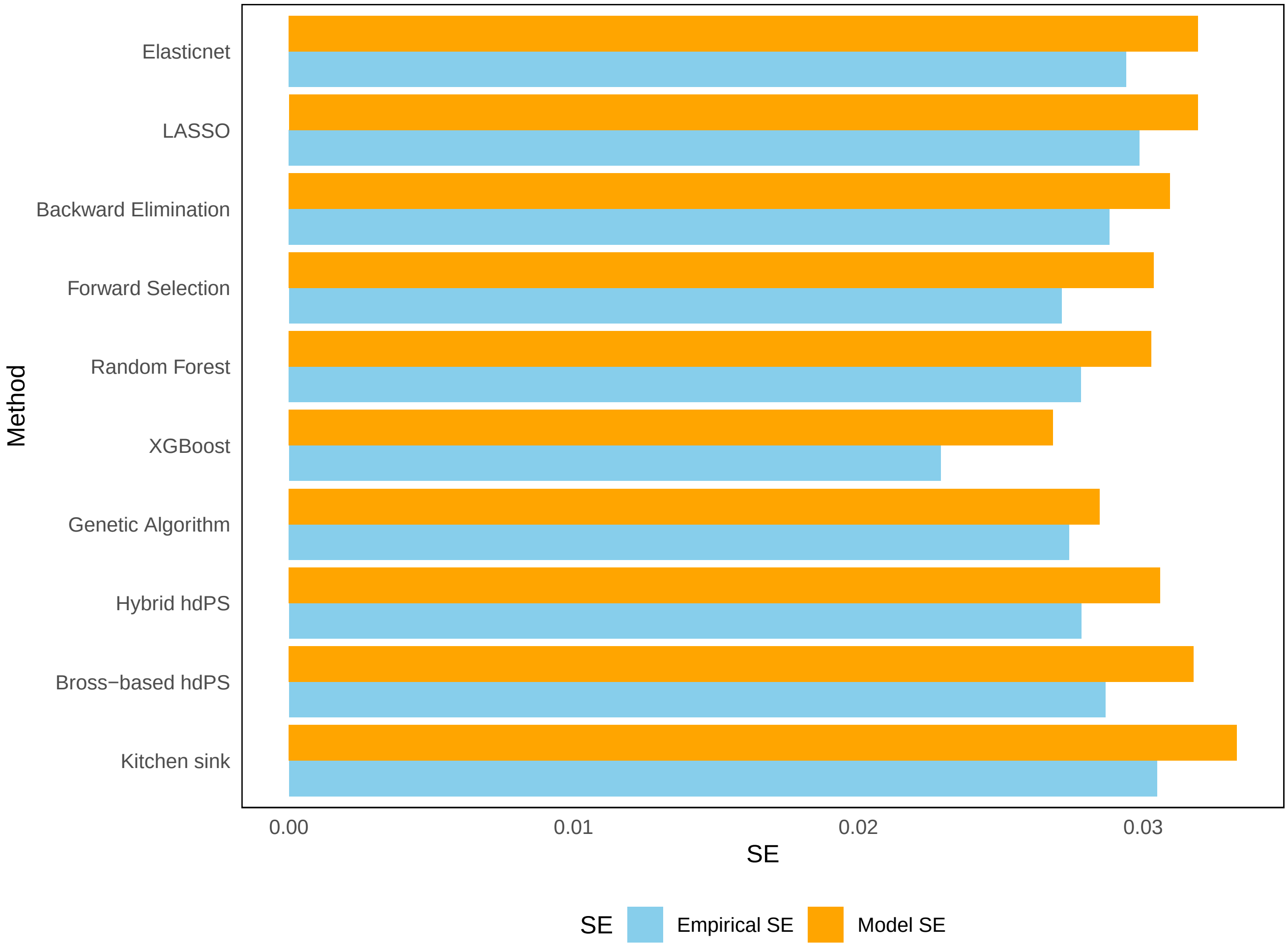

Appendix Figure C.1: Standard Error Comparison for Different Methods (Overall) when outcome and exposure are frequent.

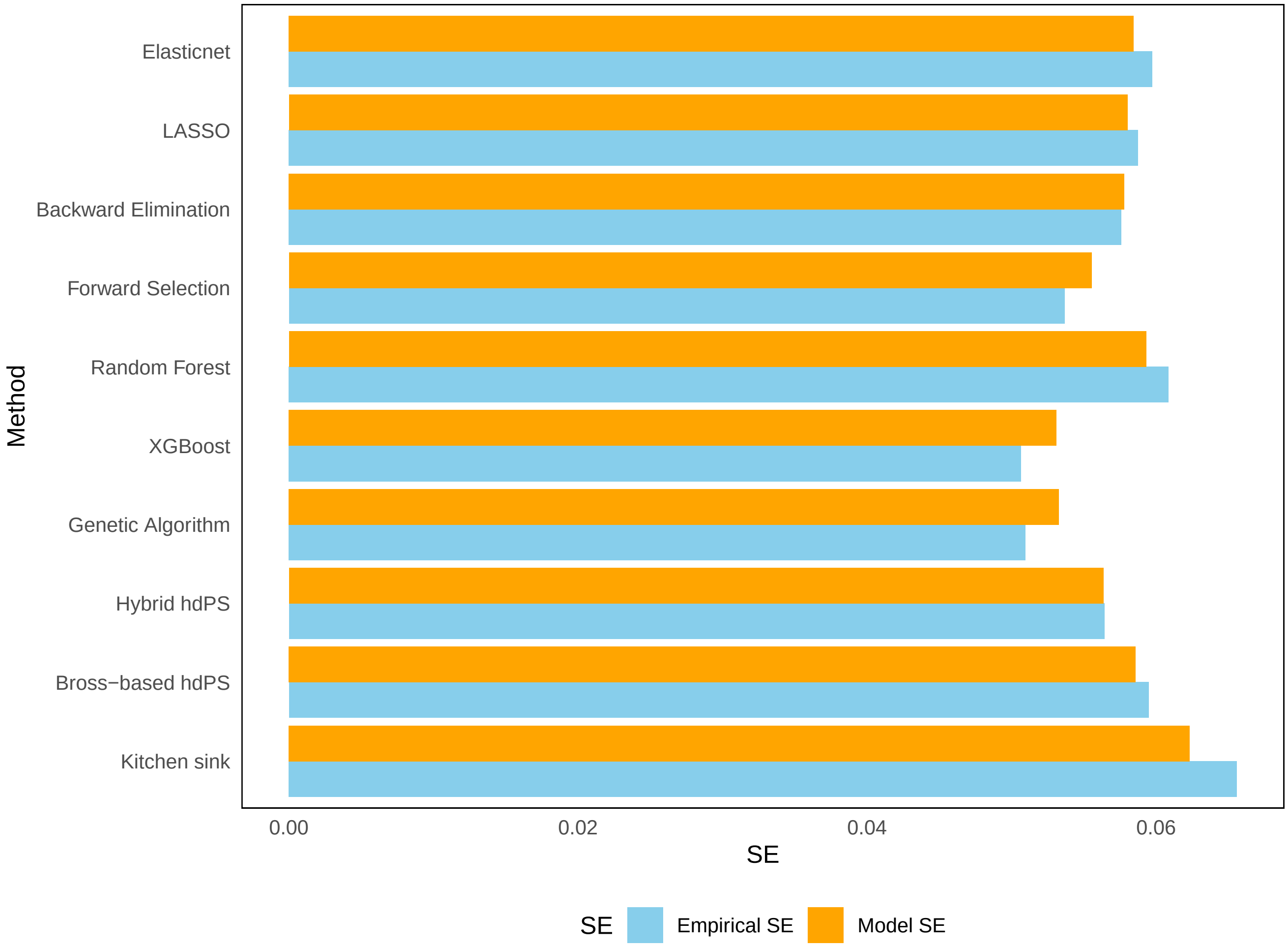

Appendix Figure C.2: Standard Error Comparison for Different Methods (Overall) when outcome is frequent but exposure is rare.

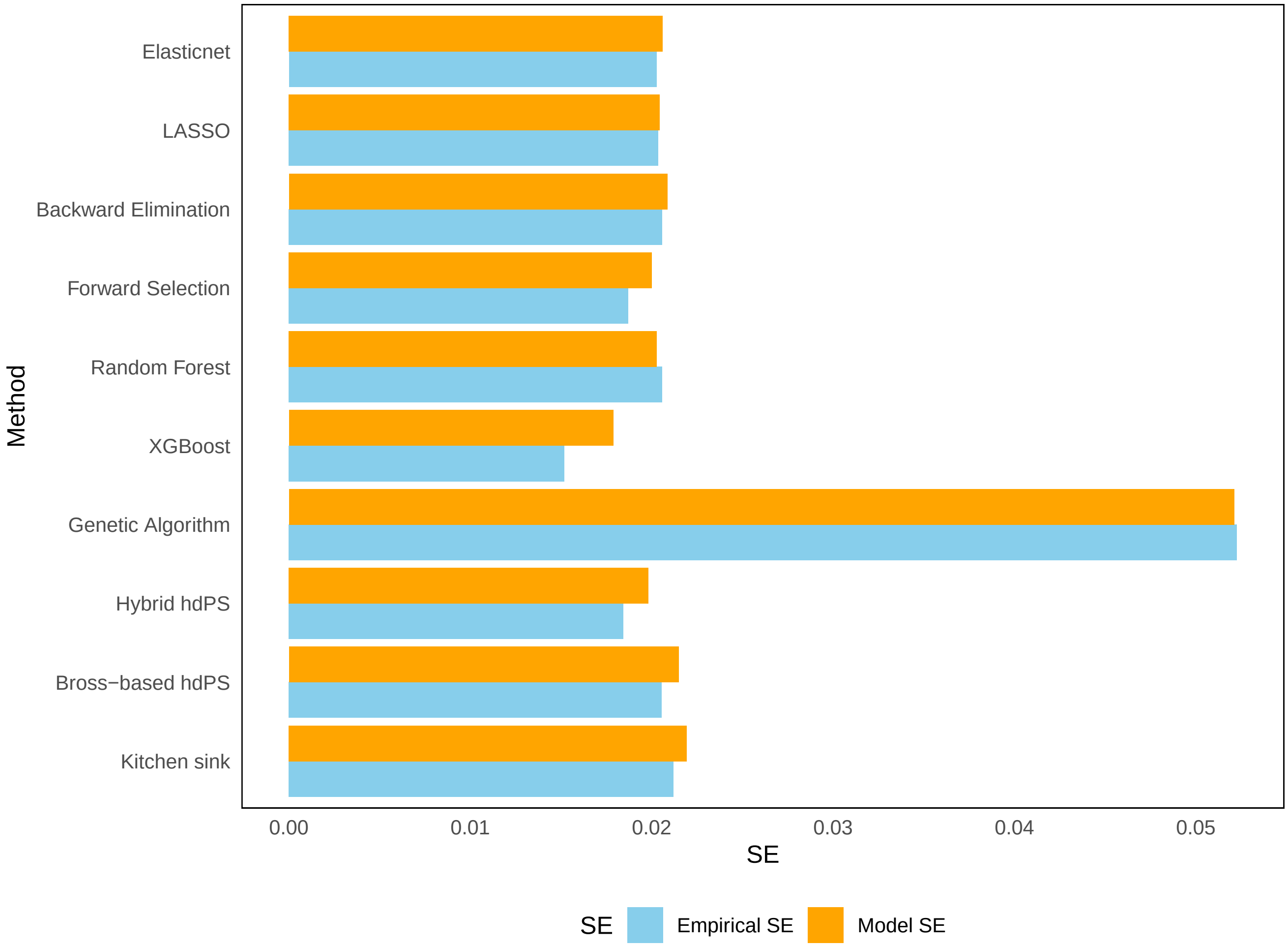

Appendix Figure C.3: Standard Error Comparison for Different Methods (Overall) when outcome is rare but exposure is frequent
