# Supplementary material for: Is there a competitive advantage to using multivariate statistical or machine learning methods over the Bross formula in the hdPS framework for bias and variance estimation?
Source: PLoS One. 2025 May 28;20(5):e0324639. doi: 10.1371/journal.pone.0324639 (PMC12118903; doi:10.1371/journal.pone.0324639)
Supplement: Attachment — Submitted filename: COMMENTS.pdf [file pone.0324639.s002.pdf]

Is There a Competitive Advantage to Using Multivariate Statistical or Machine Learning Methods Over the Bross Formula in the hdPS Framework for Bias and Variance Estimation?

COMMENTS

Overview:

The paper provides a comprehensive evaluation of various proxy selection methods in the high-dimensional propensity score (hdPS) framework. It compares traditional statistical methods with machine learning approaches to assess their performance in terms of bias, mean squared error (MSE), coverage, and standard error (SE) across different epidemiological scenarios. The study also leverages a plasmode simulation using NHANES data to draw conclusions about these methods' efficacy. The findings highlight how machine learning methods like XGBoost outperform traditional methods like the Bross formula in precision, but can incur higher bias in some scenarios. Below are the comments given for each section.

Section	Comments
Introduction and literature review	The introduction could benefit from more context on why hdPS is critical in modern epidemiology and how it directly impacts causal inference, especially in large-scale healthcare data analysis.
	Consider expanding on the limitations of traditional propensity score methods and how hdPS addresses these challenges.
	The literature review section would benefit from a clearer connection to the current study. It could briefly mention how previous work has assessed machine learning in hdPS, setting up the novelty of this study's approach.
Methods	While the paper provides an extensive methodology section, some aspects of the simulation design could be clearer. For example, explain more clearly the rationale behind selecting the specific machine learning models (e.g., Genetic Algorithm, XGBoost) and statistical methods. What makes these particular models suited for this analysis?
	The use of the "kitchen sink model" could be elaborated on. It's mentioned as a baseline but lacks a detailed description of its implications in the context of this study.
	A brief explanation of the "forward selection" and "backward elimination" methods would be beneficial to readers unfamiliar with these techniques, as they are relatively basic compared to machine learning models.
Results	While the simulation results are well-presented, additional discussion on how bias and MSE were interpreted across the different methods would help. For instance, while XGBoost shows lower MSE, the increase in bias could be further explored to contextualize the trade-offs involved.
	The explanation for why Genetic Algorithm performs poorly in terms of bias and MSE should be more detailed. Are there particular reasons why this algorithm struggles with high-dimensional data compared to others?
Table	The paper could improve the readability of Table 2 and Table 3 by providing a brief summary or interpretation of key results directly in the table captions. A clearer focus on how these results contribute to the broader argument would enhance the table's value.
Conclusions	The conclusions are robust but could be expanded to include more practical guidance for future research and applications. For example, in

	which specific types of epidemiological studies (e.g., rare diseases vs. common conditions) would one method be preferred over another?
Others questions	How can machine learning models be improved for higher bias reduction in hdPS analysis?
	What is the potential impact of using hybrid machine learning models in health data science?
	How can the results of this study be applied to real-world healthcare settings?
