# Supplementary material for: Is there a competitive advantage to using multivariate statistical or machine learning methods over the Bross formula in the hdPS framework for bias and variance estimation?
Source: PLoS One. 2025 May 28;20(5):e0324639. doi: 10.1371/journal.pone.0324639 (PMC12118903; doi:10.1371/journal.pone.0324639)
Supplement: Attachment — Submitted filename: Response to Reviewers.pdf [file pone.0324639.s003.pdf]

PONE-D-25-00468
Is There a Competitive Advantage to Using Multivariate Statistical or Machine Learning Methods Over the Bross Formula in the hdPS Framework for Bias and Variance Estimation?
PLOS ONE

Authors' response

Please find our point-by-point responses in the boxes.

Reviewer 1

**Comment from the Authors:** Reviewer 1 kindly provided both in-text comments and a set of rephrased questions in an attached PDF. For clarity and completeness, we have consolidated these comments and listed the corresponding questions below to address them collectively.

The manuscript titled "Is There a Competitive Advantage to Using Multivariate Statistical or Machine Learning Methods Over the Bross Formula in the hdPS Framework for Bias and Variance Estimation?" offers a comprehensive evaluation of various proxy selection methods within the high-dimensional propensity score (hdPS) framework. It compares traditional statistical methods with machine learning approaches, and it effectively assesses their performance across various epidemiological scenarios. The use of a plasmode simulation with NHANES data adds significant value to the study's methodology and conclusions.

Comment from attached PDF:

The paper provides a comprehensive evaluation of various proxy selection methods in the highdimensional propensity score (hdPS) framework. It compares traditional statistical methods with machine learning approaches to assess their performance in terms of bias, mean squared error (MSE), coverage, and standard error (SE) across different epidemiological scenarios. The study also leverages a plasmode simulation using NHANES data to draw conclusions about these methods' efficacy. The findings highlight how machine learning methods like XGBoost outperform traditional methods like the Bross formula in precision, but can incur higher bias in some scenarios. Below are the comments given for each section.

However, I believe that the manuscript requires major revisions to improve its quality and robustness. The following points outline the areas that need attention:

Introduction and Literature Review: The introduction could be enhanced by providing more context on the critical role of hdPS in modern epidemiology. Specifically, expanding on how hdPS addresses the limitations of traditional propensity score methods and directly impacts causal inference in healthcare data would strengthen the manuscript. The literature review could also benefit from a clearer connection to prior research, particularly focusing on how previous studies have applied machine learning to hdPS and how this study brings new insights to the field.

Comment from attached PDF:

- Introduction and literature review

1. The introduction could benefit from more context on why hdPS is critical in modern epidemiology and how it directly impacts causal inference, especially in large-scale healthcare data analysis.
2. Consider expanding on the limitations of traditional propensity score methods and how hdPS addresses these challenges.
3. The literature review section would benefit from a clearer connection to the current study. It could briefly mention how previous work has assessed machine learning in hdPS, setting up the novelty of this study's approach.

Response: In response to the reviewer's suggestion, we have revised the introduction and literature review to better contextualize the role of hdPS in modern epidemiology, clarify its advantages over traditional propensity score methods, and establish how our work builds on existing literature:

"Importantly, hdPS has become a foundational method in modern epidemiologic research that uses healthcare databases, offering a scalable and reproducible approach to confounding control when traditional investigator-specified propensity score models are limited by unmeasured, mismeasured, or omitted variables. Unlike conventional PS methods that rely solely on expert knowledge and a fixed set of covariates, hdPS empirically identifies and prioritizes proxy variables from high-dimensional data, allowing it to adjust for latent confounding structures more effectively. This makes hdPS particularly valuable for improving causal inference in observational healthcare studies, including those evaluating drug safety, treatment effectiveness, and health system interventions [5,6]."

"Several studies have proposed hybrid approaches that combine machine learning and hdPS to enhance variable selection and bias reduction while maintaining interpretability [6,7,9]. … These efforts reflect a growing interest in refining hdPS with modern statistical learning tools to optimize proxy confounder adjustment in realistic, data-rich settings."

"To extend prior research, our study systematically compares multiple machine learning and hybrid hdPS approaches using plasmode simulations grounded in real-world data structure. Importantly, we expand beyond commonly reported metrics such as bias and MSE by incorporating underutilized diagnostics—namely, empirical coverage and relative error in standard error—to provide a more nuanced understanding of estimator performance."

Methods Section: While the methodology is generally well-detailed, there is a need for further clarification regarding the choice of machine learning models (e.g., Genetic Algorithm, XGBoost) and statistical methods. A better explanation of why these particular models were selected and their relevance to hdPS would make the study's approach more transparent. The "kitchen sink model" is also mentioned briefly but needs further elaboration on its role in the analysis. Furthermore, providing a clearer explanation of the traditional variable selection methods, such as "forward selection" and "backward elimination," would benefit readers who may not be familiar with these techniques.

Comment from attached PDF:

- Methods

4. While the paper provides an extensive methodology section, some aspects of the simulation design could be clearer. For example, explain more clearly the rationale behind selecting the specific machine learning models (e.g., Genetic Algorithm, XGBoost) and statistical methods. What makes these particular models suited for this analysis?
5. The use of the "kitchen sink model" could be elaborated on. It's mentioned as a baseline but lacks a detailed description of its implications in the context of this study.
6. A brief explanation of the "forward selection" and "backward elimination" methods would be beneficial to readers unfamiliar with these techniques, as they are relatively basic compared to machine learning models.

Response: To improve transparency and clarity in our methodological choices, we elaborated on the rationale for selecting specific machine learning models, clarified the role of the kitchen sink model, and expanded explanations of traditional variable selection methods, as follows:

"The selected machine learning models were chosen for their demonstrated ability to handle high-dimensional data and perform feature selection effectively. For example, XGBoost and Random Forest are tree-based ensemble methods known for capturing non-linear relationships and interactions. Genetic Algorithm (GA) offers a stochastic, optimization-based approach to variable selection, allowing exploration of complex covariate spaces. These models align with the goals of hdPS by enabling automated, multivariate proxy variable selection that may capture complex dependencies missed by univariate selection strategies like the Bross formula. Incorporating such models expands the flexibility and potential robustness of hdPS analyses in high-dimensional data settings."

"Kitchen sink model: The kitchen sink model serves as a comprehensive baseline that includes all available covariates—both investigator-specified and proxy variables—without any variable selection. It is used to benchmark the performance of variable selection methods against a model that assumes complete adjustment [7]."

"Stepwise: Stepwise selection is a commonly used variable selection technique that builds or reduces models by adding or removing predictors based on statistical criteria—in our case, adjusted R-squared. We implemented two versions: (a) Forward selection (FS) begins with a minimal model, typically including only investigator-selected covariates. It then adds one proxy variable at a time—the one that most improves model performance at each step. This process continues until no additional variable meaningfully improves the fit. (b) Backward elimination (BE) takes the opposite approach. It starts with a full model containing all investigator-selected and proxy variables and removes the least informative variable at each step, based on its contribution to model fit. This continues until removing more variables no longer improves or begins to harm the model. These stepwise approaches are transparent, making them accessible alternatives for confounding adjustment in high-dimensional settings."

<u>Results and Discussion</u>: The simulation results are well-presented, but additional discussion on the interpretation of bias and MSE would help contextualize the trade-offs involved in using machine learning methods like XGBoost. While XGBoost shows lower MSE, the increase in bias should be explored in more detail to help readers understand the model's limitations. Similarly, the poor performance of the Genetic Algorithm should be explained with greater depth, especially regarding its struggles with high-dimensional data compared to other methods.

Comment from attached PDF:

- Results

7. While the simulation results are well-presented, additional discussion on how bias and MSE were interpreted across the different methods would help. For instance, while XGBoost shows lower MSE, the increase in bias could be further explored to contextualize the trade-offs involved.
8. The explanation for why Genetic Algorithm performs poorly in terms of bias and MSE should be more detailed. Are there particular reasons why this algorithm struggles with high-dimensional data compared to others?

Response: We expanded the discussion to clarify the interpretation of bias and MSE, particularly to contextualize trade-offs associated with models like XGBoost, and to provide a more detailed explanation of the Genetic Algorithm's limitations:

"While XGBoost consistently yielded the lowest MSE across scenarios—indicating high overall accuracy—this gain came at the cost of increased bias, particularly in rare exposure settings. This pattern suggests that while XGBoost is highly efficient in minimizing prediction error, it may over-prioritize variance reduction at the expense of bias, potentially due to aggressive regularization or suboptimal feature weighting in the context of non-linear, sparse exposure structures. In causal inference applications, such trade-offs warrant caution, as models with high precision but biased estimates can result in systematically misleading conclusions regarding treatment effects.

GA exhibited consistently high bias and MSE, indicating substantial limitations in handling the dimensionality and noise characteristics inherent in the hdPS framework. One plausible explanation is that GA's stochastic search process lacks the structure to efficiently navigate large covariate spaces with many weakly informative or correlated variables. Unlike regularized regression or tree-based methods, GA does not incorporate intrinsic mechanisms for managing multicollinearity or prioritizing marginally predictive features, which may lead to unstable or suboptimal variable subsets and subsequently poor confounding adjustment. Its computational intensity further compounds these limitations, making it less practical for high-dimensional epidemiologic analyses."

Tables and Figures: The readability of Table 2 and Table 3 could be improved by adding brief summaries or interpretations of the key results in their captions. This would help readers quickly grasp the significance of the findings and how they support the broader argument.

Comment from attached PDF:

- Table

9. The paper could improve the readability of Table 2 and Table 3 by providing a brief summary or interpretation of key results directly in the table captions. A clearer focus on how these results contribute to the broader argument would enhance the table's value.

Response: To enhance readability and aid interpretation, we revised the captions of Tables 2 and 3 to include key summaries that highlight how the results support the study's broader findings:

“Table 2: … Diagonal entries show the total number of proxies selected by each method, and off-diagonal entries represent the count of shared variables between method pairs. Most methods share a moderate number of proxies (typically 50–60 percent of the smaller set), indicating partial agreement in variable selection. Higher overlap is observed between closely related methods (e.g., LASSO and Elastic Net, or Hybrid with Bross/LASSO), while methods like XGBoost and Genetic Algorithm show lower overlap with others, reflecting divergent selection behavior in high-dimensional settings.”

“Table 3: … The Hybrid method (Bross + LASSO) shows perfect agreement with the Bross-based hdPS by design. Other methods demonstrate overlap rates ranging from 0.69 to 0.79, indicating moderate consistency in variable selection. XGBoost shows the highest overlap (79 percent) among the non-hybrid methods, while the Genetic Algorithm shows the lowest (69 percent), reflecting greater divergence in selected proxies. These overlap patterns highlight methodological differences in how each approach prioritizes covariates in high-dimensional settings.”

<u>Conclusions</u>: The conclusions are generally strong, but they could be expanded to provide more practical guidance for future research and real-world applications. Specifically, the authors could discuss which methods might be more appropriate for different types of epidemiological studies, such as those focused on rare diseases versus common conditions.

<u>Comment from attached PDF:</u>

- Conclusions

10. The conclusions are robust but could be expanded to include more practical guidance for future research and applications. For example, in which specific types of epidemiological studies (e.g., rare diseases vs. common conditions) would one method be preferred over another?

Response: In response to the request for more practical guidance, we expanded the conclusion to provide recommendations for method selection across different epidemiological study contexts:

“These findings have practical implications for selecting variable selection strategies in epidemiological research. For studies focused on rare exposures (e.g., occupational or genetic risk factors), methods that minimize bias—such as Bross-based hdPS, Hybrid hdPS, or forward/backward stepwise selection—may be more suitable, as they provide more stable effect estimates under sparse data conditions. In contrast, studies targeting common conditions or aiming to improve precision, such as evaluations of widely used treatments or lifestyle exposures, may benefit from machine learning method such as XGBoost, which consistently demonstrate lower MSE. However, trade-offs between bias and variance should be carefully considered in relation to the study’s primary inference goal.”

“Finally, future simulation studies should focus on evaluating coverage and other metrics in epidemiological scenarios such as time-varying exposures, multiple treatment settings, and data with substantial measurement error, which would provide valuable insights into the generalizability of our findings [33].”

<u>Comment from attached PDF:</u>

- Others questions

11. How can machine learning models be improved for higher bias reduction in hdPS analysis?
12. What is the potential impact of using hybrid machine learning models in health data science?
13. How can the results of this study be applied to real-world healthcare settings?

Response: To address the reviewer's broader questions regarding the implications of our findings, we added the following discussion on improving machine learning models for bias reduction, the potential impact of hybrid models, and real-world applications:

"One avenue for improving bias reduction in machine learning models within hdPS analysis is the incorporation of causal structure or prior knowledge into variable selection. Methods such as targeted maximum likelihood estimation, double machine learning, or feature engineering based on directed acyclic graphs can help align machine learning-based selection with the underlying causal model. Additionally, tuning hyperparameters to favor less aggressive regularization or ensemble weighting may reduce over-smoothing and improve bias performance, particularly in low-prevalence strata.

Hybrid machine learning models—such as combinations of domain-informed methods (e.g., Bross or investigator-specified variables) with data-driven algorithms like LASSO or Random Forest—offer a promising balance between interpretability and predictive accuracy. Their application in health data science could enable more robust causal inference while accommodating real-world data challenges such as missingness, nonlinearity, and high dimensionality. Such models may help mitigate confounding bias without discarding variables of known clinical relevance, improving both validity and stakeholder trust in observational research findings."

"In real-world healthcare settings—such as electronic health record-based cohort studies, claims data analysis, or comparative effectiveness research—these results offer concrete guidance on choosing hdPS-compatible variable selection strategies. For instance, institutions conducting routine surveillance or drug safety studies may benefit from hybrid or regularized regression approaches that balance scalability with confounding control. Meanwhile, traditional stepwise methods remain attractive for smaller studies or settings requiring transparent modeling. Overall, aligning method choice with data availability, computational resources, and inferential goals can improve the practical utility of hdPS analyses in healthcare."

These revisions are essential to enhancing the manuscript's clarity, depth, and impact. A more robust discussion of the trade-offs between methods, along with clearer explanations of model choices, will help solidify the paper's contribution to the field.

Recommendation: Given the importance of the topic, I recommend that the authors address the major revisions outlined above. Once these revisions are made, I believe the manuscript could make a valuable contribution to the literature on hdPS and its applications in health data science.

Thank you for considering my review.

Reviewer 2

The manuscript presents a highly relevant comparison of hdPS variable selection methods, including both traditional statistical and machine learning approaches. The findings are useful for epidemiologists and health data scientists.

Key suggestions for revision:

- Clarify the rationale behind selecting 100 proxy variables in hdPS methods.

Response: To address the reviewer's request, we clarified why 100 proxy variables were selected in the hdPS and related methods:

"This threshold of 100 proxy variables has been used in the literature to balance model complexity and overfitting risk, while ensuring that a sufficient number of proxies are included to capture unmeasured confounding [22].Our choice reflects a practical trade-off between dimensionality and computational feasibility."

- Expand the discussion on bias correction for coverage probability.

Response: We expanded the discussion to explain how bias correction influenced the interpretation of coverage probability:

"Bias-corrected coverage provides a more accurate reflection of model reliability when bias is present. By adjusting for bias, we isolate the coverage performance attributable to variance estimation alone, allowing clearer comparisons of uncertainty calibration. This correction is crucial for distinguishing methods that produce narrow but biased intervals from those that offer valid inference under realistic misspecification."

- Address concerns regarding MSE interpretation when variance estimation is unstable.

Response: In response to the reviewer's concern about MSE interpretation, we added the following clarification:

"While MSE is a commonly used metric, its interpretation becomes less reliable when variance estimates are unstable, as observed with singly robust machine learning models. In such cases, MSE may mask the trade-off between high variance and low bias (or vice versa), especially when confidence intervals are miscalibrated. Our inclusion of coverage and standard error diagnostics addresses this limitation by providing complementary insights into inference accuracy."

- Discuss the practical implications of computational efficiency for real-world applications.

Response: We have now added a discussion on the practical importance of computational efficiency in applied settings:

"In applied research, particularly with large-scale electronic health records or claims data, computational efficiency is a critical consideration. While tree-based or stochastic optimization

methods may offer performance gains, their longer runtime may be impractical in settings requiring rapid analyses or model updates. Simpler approaches like stepwise selection or penalized regression offer viable alternatives when speed, transparency, or reproducibility are prioritized.”

- Improve clarity and conciseness in the Results and Discussion sections.

Response: We appreciate the suggestion. In line with the other specific comments, we improved clarity by adding interpretive text to the Discussion section (e.g., explaining potential reasons for the direction of results across different methods, model limitations, practical implications, future research directions, and conclusions). These additions aim to enhance readability and help readers more easily interpret the results. All changes are marked in red in the revised manuscript. Given the diversity of simulation scenarios and performance metrics considered, reported results explain the nuance, and comparability of the findings. We hope these revisions adequately address the reviewer’s comment.
