# Supplementary material for: Is there a competitive advantage to using multivariate statistical or machine learning methods over the Bross formula in the hdPS framework for bias and variance estimation?
Source: PLoS One. 2025 May 28;20(5):e0324639. doi: 10.1371/journal.pone.0324639 (PMC12118903; doi:10.1371/journal.pone.0324639)
Supplement: Attachment — Submitted filename: COMMENTS AND CONCLUSIONS.pdf [file pone.0324639.s004.pdf]

Is There a Competitive Advantage to Using Multivariate Statistical or Machine Learning Methods Over the Bross Formula in the hdPS Framework for Bias and Variance Estimation?

PONE-D-25-00468_R1_reviewer.pdf

Section	Comment Summary	Author's Response (Page 31–52)	Conclusion
**Introduction and Literature Review**	1. Expand on why hdPS is critical and how it impacts causal inference.<br>2. Discuss traditional PS limitations and hdPS improvements.<br>3. Connect past ML work to this study.	Fully addressed. The introduction clearly explains the importance of hdPS in modern epidemiology, causal inference, and large healthcare data. Traditional PS limitations and hdPS improvements are discussed (page 31–33). Also, machine learning work in hdPS is linked to the novelty of this study.	Addressed
**Methods**	1. Clarify why selected ML models (GA, XGBoost, etc.) are appropriate.<br>2. Elaborate on "kitchen sink model."<br>3. Briefly explain forward selection and backward elimination.	Addressed in detail. The rationale for selecting GA, XGBoost, Random Forest, etc., is clearly provided (page 36–37). The "kitchen sink" model is explained as a no-selection baseline model (page 36). Forward and backward selection are described understandably for non-ML readers (page 38).	Addressed
**Results**	1. Deeper interpretation of bias and MSE trade-offs.<br>2. Explain why GA performs poorly.	Addressed. The Results and Discussion sections (page 40–46) analyze how XGBoost has lower MSE but higher bias, and they explain GA's poor bias/MSE performance due to its stochastic search inefficiency in high-dimensional/noisy data settings.	Addressed

**Tables (Table 2 & 3)**	Improve table captions by interpreting results briefly.	Done. The captions of Table 2 and Table 3 now include clear summaries (page 45–46), stating how variable overlap affects conclusions about method similarities and divergence.	Addressed
**Conclusions**	Provide more practical guidance for real-world use (e.g., rare disease studies).	Done. Practical implications are provided (page 46–47), recommending different methods for rare exposure vs. frequent outcome studies, and guidance based on computational resources.	Addressed
**Other Questions**	- How to improve ML models for bias reduction?<br>- Potential impact of hybrid models?<br>- Application to real healthcare settings?	Fully discussed in "Future Direction" section (page 47–49). The authors propose ideas like incorporating causal structures into ML models, using hybrid models for better balance, and applying results in real-world datasets like EHRs and claims data.	Addressed
